# Using a self-directed workbook to support advance care planning with long term care home residents

**DOI:** 10.1186/s12904-021-00815-1

**Published:** 2021-07-29

**Authors:** Tamara Sussman, Sharon Kaasalainen, Jack Lawrence, Paulette V. Hunter, Valerie Bourgeois-Guerin, Michelle Howard

**Affiliations:** 1 McGill University School of Social Work, 3506 University St. #300, Montreal, QC H3A 2A7 Canada; 2 Faculty of Health Sciences, School of Nursing, Health Sciences Centre, McMaster University, 1280 Main Street West, Hamilton, ON 2J20L8S 4K1 Canada; 3 University of Saskatchewan, St Thomas More College, Psychology, 9 Campus Drive, Saskatoon, SK S7N 5A5 Canada; 4 Department of Psychology, University of Quebec in Montreal, 100 Sherbrook St West, Montreal, QC H3C 3P8 Canada; 5 Department of Family Medicine, David Braley Health Sciences Centre, McMaster University, 100 Main Street West, 5th floor, Hamilton, On L8P 1H6 Canada

**Keywords:** Long-term care, End-of-life communication, Advance care planning, Palliative care

## Abstract

**Background:**

While advance care planning (ACP) has been shown to improve the quality of end-of-life (EOL) communication and palliative care, it is rarely practiced in long term care (LTC) homes, where staff time to support the process is limited. This study examines the potential of a publicly available self-directed ACP workbook distributed to LTC residents to encourage ACP reflection and communication.

**Methods:**

Recruitment took place across three LTC homes, between June 2018 and July 2019. To be eligible, residents had to have medical stability, cognitive capacity, and English literacy. The study employed a mixed methods concurrent design using the combination of ranked (quantitative) and open (qualitative) workbook responses to examine documented care preferences and ACP reflections and communications.

**Results:**

58 residents initially agreed to participate in the study of which 44 completed self-directed ACP workbooks. Our combined quantitative and qualitative results suggested that the workbooks supported the elicitation of a range of resident care preferences of relevance for EOL care planning and decision making. For example, ranked data highlighted that most residents want to remain involved in decisions pertaining to their care (70%), even though less than half expect their wishes to be applied without discretion (48%). Ranked data further revealed many residents value quality of life over quantity of life (55%) but a sizable minority are concerned they will not receive enough care at EOL (20%). Open comments affirmed and expanded on ranked data by capturing care preferences not explored in the ranked data such as preferences around spiritual care and post mortem planning. Analysis of all open comments also suggested that while the workbook elicited many reflections that could be readily communicated to family/friends or staff, evidence that conversations had occurred was less evident in recorded workbook responses.

**Conclusions:**

ACP workbooks may be useful for supporting the elicitation of resident care preferences and concerns in LTC. Developing follow up protocols wherein residents are supported in communicating their workbook responses to families/friends and staff may be a critical next step in improving ACP engagement in LTC. Such protocols would require staff training and an organizational culture that empowers staff at all levels to engage in follow up conversations with residents.

## Background

People aging with chronic progressive conditions such as dementia, heart disease, or frailty may eventually experience restricted capacity to make care decisions. If communication about care preferences has not occurred, these circumstances can result in care that does not align with a person’s wishes, including invasive treatment at end-of-life (EOL) and traumatic death [1]. Further, legally appointed substitute decision-makers (SDMs) who have limited knowledge of their family member’s/friend’s preferences may feel burdened when making care decisions on their behalf [2]. These concerns are an urgent priority in long-term care (LTC), where many residents experience the deterioration of their cognitive capacity due to progressive, non-reversible health conditions [3]. Advance care planning (ACP) addresses these concerns by guiding people to reflect on, discuss, and sometimes document their values, beliefs, and preferences for future care while they still can [4, 5]. When enacted, ACP improves concordance between patients' and family/friends' preferred EOL wishes and family satisfaction with care while reducing family stress, anxiety, and depression [6, 7].

Despite its benefits, ACP is rarely practiced in LTC [8, 9]. Key barriers include lack of resident and family readiness [9], limited staff time and training [10], and uncertainty from all parties regarding the scope of ACP and when to introduce it [4, 11, 12]. For example, staff in LTC frequently express a desire for direction on how to gauge resident readiness and what to discuss beyond single medical interventions (e.g. - do not resuscitate).

The Transtheoretical Model of Change has been drawn upon by some scholars to help clinicians navigate varying degrees of readiness and expand understandings of scope [13]. For example, Sudore and colleagues et al. [14] outlined a five-stage model for ACP engagement ranging from *precontemplation*, where awareness of ACP’s importance is lacking and desire for engagement is limited; to *maintenance*, where communicated preferences are adapted to changing life circumstances. This phased holistic approach was developed to support an understanding of ACP that extends beyond single medical decisions; promotes ongoing conversations and reflections; and highlights the importance of selecting and preparing SDMs [15, 16].

Such staged-models address some key barriers noted to challenge ACP engagement in LTC. For example, they provide direction for how clinicians may recognize different levels of readiness and respond accordingly. However, the presumption that clinical staff are available to facilitate an ongoing ACP process of reflection and dialogue stands in stark contrast to the realities of LTC [17, 18] where between 70–90% of the hands-on care in LTC is provided by non-regulated staff such as personal support workers/care aides and recreational staff [18, 19]. Hence, ACP resources that encourage movement through the ACP process without a heavy reliance on clinical or regulated staff facilitation may improve ACP uptake in LTC [17].

In recent years, interactive tools, like workbooks, have been developed to encourage ACP reflections and communication with family/friends and healthcare providers [20]. While the content of these tools typically aligns with staged models of ACP engagement, their self-use design makes then less reliant on clinician facilitation. Hence LTC staff view such materials as both acceptable and feasible for use in LTC (see Sussman and colleagues for examples of self-directed tools viewed as feasible for a LTC context) [21]. However, the extent to which these self-directed tools can facilitate ACP engagement amongst LTC residents has yet to be explored.

Addressing this important gap in the literature, our study examined the scope of future care preferences elicited by a self-directed ACP workbook, called Your Conversation Starter Kit, when distributed amongst residents in LTC. The study further explored the extent to which the workbook elicited two key aspects of ACP engagement: reflection and communication. More specifically our study aimed to answer the following research questions:

1. What types of future care preferences do persons in LTC express within a self-directed ACP workbook?
2. Does such a workbook activate future care reflection and communication between residents and families/SDMs?

## Methods

This study used a concurrent mixed-method design to meet its aims. Our analysis of care preferences (research question one) was informed by both open- and closed-ended workbook responses. Complementing closed-ended responses with open-ended comments allowed us to elaborate on and clarify the expressed preferences [22]. Our analysis of ACP reflection and communication (research question two) was informed by open-ended workbook responses. Data were drawn from residents living in three LTC homes in urban Southern Ontario, Canada that had participated in a larger study aimed at improving engagement in ACP in LTC [23].

### Sampling & Recruitment

Eligibility for the larger study included: (1) medical stability, assessed by a score of three or lower using the Changes in Health and End-Stage disease and Symptoms and Signs (CHESS) calculation [24], and (2) cognitive capacity for healthcare decision-making, assessed by the judgement of clinical LTC home staff who were on-site leaders for the project, and (3) English literacy for oral and written communication. Willingness and cognitive capacity to participate was monitored by the research team on an ongoing basis over the course of the intervention. This was done by observing residents’ capacities to respond to questions appropriately, ask for clarity when needed, and pose questions evidencing an understanding of the study and the intervention [25]. These criteria excluded persons whose cognitive capacity had deteriorated to a stage wherein ACP was no longer feasible. Eligibility for the current study included (4) use of the ACP workbook.

Recruitment for the larger study took place over the course of a year, between June 2018 and extended until July 2019. Residents who met eligibility criteria were approached by staff about their participation. Interested residents were contacted by research staff, and an in-person visit was arranged to review the purpose of the study and expectations of involvement. If participants confirmed agreement, written informed consent was attained. Based on profiles of residents known to reside in Canadian LTC homes we expect that approximately 31% of those residing in our participating homes (153/495) met eligibility requirements for the larger study [26].

### Intervention

The workbook was selected because it had several features found to support tool usability in LTC, including: information on the importance of ACP and family/friend roles; reflective prompts on personal values and care preferences; guiding questions to support communication; a self-directed paper-based format; and a moderate 12-page length [22]. Following enrollment, participants were given a brief orientation to the workbook and ACP by research staff. We elected to simply distribute the workbook and see how, if at all, residents engaged with it, as the sustainability of ACP programs in LTC has been an issue when interventions are heavily reliant on staff facilitation [27]. Participants were given one month to read the workbook, enter their responses, and have relevant discussions with family/friends. Participants’ workbook responses (or lack thereof) were the main source of data in our study.

### Data Collection

#### Participant Profiles

At enrollment, all participants were given a questionnaire that asked their age, gender, race/ethnicity, religion, marital status, education, and length of time residing in LTC. Health conditions and CHESS scores were collected from participants’ medical charts.

#### Future Care Values and Preferences

Participants’ responses to the workbook’s nine five-point Likert-type questions and 12 open-ended questions captured their care preferences. The open-ended questions can be found in Table 1, and the Likert-type questions are embedded in Table 2 alongside tallied responses.

**Table 1:** The ACP workbook's 12 open-ended questions

Step	ACP Stage	Open-ended questions
1	Precontemplation	What do you need to do before you feel ready to have the conversation? Do you have any particular concerns that you want to be sure to talk about?
2	Contemplation	What matters to me through the end-of-life is… What kind of role do you want to have in the decision-making process? What do you notice about the kind of care you want to receive? What role do you want your loved ones to play? What do you feel are the three most important things that you want your friends, family, and/or health care team to understand about your wishes and preferences for end-of-life care?
3	Preparation	What do you want to be sure to say?
4	Reflection on Conversations	Is there something you need to clarify that you feel was misunderstood or misinterpreted? Who do you want to talk to next time? Are there people who should hear things at the same time? How did this conversation make you feel? What do you want you/your loved ones to remember? What do you want to make sure to ask or talk about next time?

**Table 2:** Future Care Values and Preferences; Closed Ended Workbook Responses

	**Ranked Questions**	**N (%)**
**Decision-Making**	As a patient I’d like to know ___ about my condition and treatment.	
	The basics	6 (14%)
	Mixed preferences	3 (7%)
	The details	33 (75%)
	If I had a terminal illness, I would prefer to ___.	
	Not know how quickly it is progressing	16 (36%)
	Mixed preferences	4 (9%)
	Know my doctor’s best estimation for how long I have to live	23 (52%)
	As I receive care, I would like ___.	
	My health care team to do what they think is best	8 (18%)
	Mixed preferences	3 (7%)
	To have a say in every health care decision	31 (70%)
**Family/Friend Involvement**	How involved do you want your loved ones to be?	
	Do exactly what I’ve said, even if it makes them uncomfortable	21 (48%)
	Mixed preferences	9 (20%)
	Do what brings them peace, even if it goes against what I’ve said	10 (23%)
	When it comes to your privacy [at death], ___.	
	When the time comes, I want to be alone	7 (16%)
	Mixed preferences	8 (18%)
	I want to be surrounded by my loved ones	25 (57%)
	When it comes to sharing information about my illness with others, ___.	
	I don’t want my loved ones to know everything	4 (9%)
	Mixed preferences	5 (11%)
	I am comfortable with loved ones knowing everything	32 (73%)
**Treatment Care**	How much medical treatment do you want to try?	
	Every treatment available, no matter how uncomfortable I become	9 (20%)
	Mixed preferences	8 (18%)
	Quality of life is more important to me than quantity	24 (55%)
	What are your concerns about medical treatments?	
	I worry that I won’t get enough care	9 (20%)
	Mixed concerns	29 (66%)
	I worry that I’ll get too much care	3 (7%)
	What are your preferences about where you want to be?	
	I want to spend my last days in a health care facility	14 (32%)
	Mixed preferences	5 (11%)
	I want to spend my last days at home	21 (48%)

#### Future Care Reflections and Communication

Participants’ responses to the 12 open-ended questions also captured the extent to which they engaged in ACP reflections and communication with family/friends and staff.

### Data Analysis

#### Quantitative Analysis

Prior to descriptive analysis, all five-point Likert-type responses were transformed to a three-point scale to facilitate data presentation. For example, potential responses to the workbook question about the extent to which participants wish to be informed about their health conditions ranged from “only the basics” (1) to “all the details” (5). Workbook ratings of 2, 3 and 4 were not given specific anchors. Consequently, we reclassified ratings such that 1 – 2 became 1 (preference for low information), 3 became 2 (preference for a moderate amount of information), and 4 – 5 became 3 (preference for detailed information). Frequencies and ranges were generated for all demographic (e.g., age, racial identity), health (e.g., reported conditions; CHESS scores), and workbook data to capture participants’ profiles and expressed preferences.

#### Qualitative Analysis

All workbook responses to open-ended questions were photocopied, transcribed verbatim and imported into a qualitative analysis program called Dedoose [28]. We conducted a three-stage directed (i.e., deductive) content analysis of the responses to examine both types of care preferences (*ACP content*) and the extent to which participants revealed apprehensions, reflections, and communications (*ACP process*) [29].

In the first stage of analysis, the first and third authors engaged in ‘data familiarization’ (i.e., a full and general reading of the data), where we noticed many responses to open-ended questions aligned with issues addressed in the Likert-type questions. For example, a Likert-type question asked participants their preference for active treatment versus a focus on quality of life; a theme frequently elaborated on in open-ended responses. Hence, we created an eight-code structure for *ACP content* broadly based on the content areas explored in the Likert-type questions (e.g., being informed, location of death, and decision-making involvement). These content areas also reflect well-documented concerns to older persons [30–32]. Data that fell outside the eight-code structure were coded inductively. That is, we assumed that the scope of participants’ concerns would extend beyond those prompted in the workbook such that additional content codes would ultimately emerge. To analyze *ACP process*, we created a four-code structure based on Sudore and colleagues' [14] model of ACP engagement: pre-contemplation (all expressions of apprehension, uncertainty, or disinterest in ACP); contemplation (all expressions of care preferences); readiness for action (all expressions of intent to communicate or discuss ACP with others); and reflections on action (all expressions that communication or reflection thereof had occurred).

In the second stage, the third author applied the initial coding structure to the data excerpts. Each excerpt was assigned both an *ACP content* code and an *ACP process* code. Both analysts discussed all excerpts that aligned with multiple elements of the coding structure, did not easily align with any elements, or were otherwise difficult to code. Finally, both authors reviewed a random sample of approximately 10% of the excerpts (55/557), safeguarding the credibility and comprehensiveness of the analysis [33]. By the end of this second stage, we had developed 11 *ACP content* codes (i.e., eight directed by the workbook and three emergent).

In the third and final stage, we collapsed the 11 *ACP content* codes into six meaningful higher-order categories: three based on the deductive coding scheme and three emergent. The *ACP process* codes were also collapsed into meaningful higher-order categories. We agreed that the pre-contemplation category included many excerpts that might reflect other processes (e.g., a response of ‘not applicable’ might represent apprehension, or that reflection already occurred). Additionally, we felt it was important to capture how the framing of participants’ reflections ranged from vague to clear, as value clarification is a critical step in the ACP process [33]. Hence, we created four *ACP process* categories: *vague reflection*, contemplations that require further clarification to inform potential decisions (e.g., ‘I want comfort’); *specific reflection*, contemplations that clearly translate to actions (e.g., ‘I do not want to be in-and-out of the hospital at EOL’); *preparing for communication*, expressions of plans to discuss specific topics or to talk to specific people; and *reflection on communication*, expressions of thoughts and feelings about a previous conversation (e.g., ‘I felt good after the conversation’).

Finally, we tallied the number of participants who made at least one statements within each category. All participants were ascribed pseudonyms to maintain confidentiality while allowing their personal thoughts and experiences to be followed. The evolution of the *ACP content* and *ACP process* coding structure is summarized in Table 3 and Table 4.

**Table 3:** Content Code Evolution: Care Preferences

	**Excerpts**	**Codes (*Sentiment*)**	**Categories**
**Directed**	"I want to know all about my condition and treatment process" (*“John”, 87*)	Being Informed (*All the details*)	Decision Making
	"I would prefer that I have control in decision making process" (*“Brett”, 52*)	Shared Decisions: Health Team (A *say in every decision*)	
	"Most my family know what I want. Respect my decision. Go along with my decisions." (*“Jim”, 76*)	SDM Flexibility (*Do exactly what I’ve said*)	Family/Friend Involvement
	"I want my family around me, soft classical music. Have comfortable area for my family around me." (*“Geoff”, 86*)	Bedside Company (*Surrounded by loved ones*)	
	"It is important that my family is aware of my health condition." (*“Julie”, 69*)	Sharing Information (*Know everything*)	
	"Quality is more important than quantity of time" (*“Bette”, 92*)	Quantity vs Quality of Life (*Quality* > *Quantity*)	Treatment Care
	"I remember my husband's EOL and I don't want to be in that situation, in and out of the hospital 3 times and it was unpleasant" (*“Becca”, 82*)	Treatment Care (*Overly aggressive care*)	
	"I want it to be pain free and want to be at home at the end-of-life" (*“John”, 87*)	Death Location (*At home/LTC*)	
**Emergent**	"I just want to thank them for doing their best to help me" (*“Martha”, 68*)	Saying Thank-You	Interpersonal Communication
	"Saying good bye to the ones that I love" (*“Ellen”, 88*)	Saying Good-bye	
	"last rights" (*“Mary”, 76*)	Deathbed Religious Rituals	Religious & Cultural
	"[I want to be sure to say] that I am leaving this life here to be with my Saviour, Jesus, who loves and cares for us all." (*“Luke”, 88*)	Valuing Heaven	
	"making sure my will is in order and my finances will go to my oldest son for emergencies" (*“Jane”, 70*)	Estate Planning	Post-Mortem Preparations
	"No memorial service" (*“Stuart”, 81*)	Funeral Planning

**Table 4:** Process Code Evolution: ACP Reflection and Communication

	**Excerpts**	**Preliminary Categories**	**Categories**
**ACP Engagement Process**	“[my thoughts before having the conversation are] nothing in particular”	Precontemplation	
	“[I want my family and friends to be] involved”	Contemplation	Vague Reflection
	“I want excellent care. I want treatments that are not too uncomfortable. I want to live out the rest of my days happy and peaceful”		Specific Reflection
	“[what matters most is that I] talk to family members”	Readiness for Communication	Readiness for Communication
	“[next time,] I would like to continue the conversation with my sister”	Reflection on Communication	Reflection on Communication

## Results

### Quantitative Results

#### Participant Profiles

A total of 58 residents across study sites were approached by staff and all agreed to hear more about the study. After a member of the research team followed up, 55 residents consented to participate and received an ACP workbook. Of these, 44 residents returned their workbooks to research staff and are thus included in the sample for this study. Table 5 provides an overview of the profiles of enrolled participants. Our sample was almost evenly split between men (43%, 19/44) and women (57%, 25/44). Most were White (80%, 35/44) and older than 75 (66%, 29/44; $\overline{x }$ = 78, SD = 10). Education level varied considerably. While more than half of the sample (55%, 24/44) attained some form of post secondary education (e.g. a university level bachelor’s degree), more than a third (34%, 15/44) did not complete secondary school. Almost half reported at least two health conditions. The most frequently reported health conditions were diabetes (41%, 18/44), dementia (39%, 17/44), and heart disease (39%, 17/44). All participants had a CHESS score indicative of low to no health instability (100%. 44/44), and most had lived in LTC for at least one year (57%, 25/44).

**Table 5:** Participant (*N*=44) profiles, given in freq (%)

**Sex**	Female	25 (56.8%)	**Education**	Post secondary	24 (54.5%)
	Male	19 (43.2%)		No secondary school	15 (34.1%)
**Age**	85+	16 (36.4%)		Secondary school	5 (11.4%)
	75-84	13 (29.5%)	**Health Conditions**	Diabetes	18 (40.9%)
	65-74	10 (22.7%)		Dementia	17 (38.6%)
	0-64	4 (9.1%)		Heart disease	17 (38.6%)
**Race**	White	35 (79.5%)		Respiratory disease	10 (22.7%)
	Racialized	9 (20.5%)		Cancer	5 (11.4%)
**Marital Status**	Widowed	19 (43.2%)		Kidney disease	1 (2.3%)
	Never married	10 (22.7%)		2 of the above	21 (47.7%)
	Married/common-law	7 (15.9%)		3 of the above	9 (20.5%)
	Divorced/separated	7 (15.9%)	**CHESS Score**	0	23 (52.3%)
	Prefer not to answer	1 (2.3%)		1	18 (40.9%)
**Religion**	Christian	29 (65.9%)		2	2 (4.5%)
	No religion	8 (18.2%)	**Years in LTC**	0-1	15 (34.1%)
	Jewish	6 (13.6%)		1-3	12 (27.3%)
	Other	1 (2.3%)		3+	13 (29.5%)

#### Future Care Preferences: Closed-Ended Workbook Responses:

Table 2 provides an overview of participants’ responses to all Likert-type questions. The following is a summary of key trends regarding care preferences and preferred decisional involvement.

##### Decision-Making Preferences

Most respondents wanted to know all the details about their condition (75%, 33/44), and to have a say in every medical decision (70%, 31/44).

##### Family/Friend Involvement

Most respondents were comfortable with their family/friend knowing everything about their health conditions (73%, 32/44). Many wanted family/friends to follow directives exactly in the event of incapacity (48%, 21/44), but sizable minorities were either uncertain (20%, 9/44) or comfortable with deviations, especially if adherence to directives would cause family/friends undue burden or suffering (23%, 10/44).

##### Treatment Care Preferences

Most respondents valued quality of life over quantity (55%, 24/44), but some wanted to receive care indefinitely (20%, 9/44) and were concerned that they may not receive enough care (20%, 9/44). While many indicated a preference to die at home (48%, 21/44), some preferred to spend their EOL in a health care facility (32%, 14/44). Most respondents wanted to be surrounded by friends/family at EOL (57%, 25/44).

### Qualitative Results

#### Future Care Values and Preferences: Open-Ended Workbook Responses

An overview of the six content categories and their associated open-ended responses is included Table 6.

**Table 6:** Future Care Values and Preferences: Open Ended Workbook Responses

	**Category**	**n**	**Excerpt Examples**
**Directed**	Decision Making	39	"I would prefer that I have control in decision making process" “[I’d prefer to] be aware of everything but [I] trust the doctors’ [decisions]” “If I lose my memory or I am getting there, I would want to be taken out of the decision-making process”
	Family/Friend Involvement	39	“[my SDM] is my only family. I want her to be the only one involved. She’s been along for the whole ride. She knows exactly what I want; sometimes she knows more than I do.” “My family members may know about my disease and health condition and I am comfortable if they stay beside me at my last hours” “Most of my family know what I want. Respect my decision. Go along with my decisions”
	Treatment Care	41	“Having someone here at the nursing home with compassion and understanding, not just a worker following a health care book and doing what they choose to do to you” “If I am young and dying, I want full treatment; if I am old, than I am okay with comfort care” “I don’t want to suffer”
**Emergent**	Interpersonal Communications	14	“I just want to thank [my loved ones] for doing their best to help me” “[I want to be sure that] everyone’s looked after. No fighting/no arguments” “[What matters most is] saying good bye to the ones that I love”
	Religious & Cultural	6	“[one of my most important EOL preferences is that] I want a pastor to recite the bible” “Make sure my cultural wishes are followed: no autopsy, no cremation, must be in my native clothing” “I believe in life after death. No blood transfusion”
	Post-Mortem Preparations	26	“making sure my will is in order and my finances will go to my oldest son for emergencies” “Any body part that’s good take it (organ donation)” “I want to be cremated”

##### Decision-Making Preferences

Most respondents made at least one open-ended comment in support of decisional involvement (89%, 39/44). Affirming the trend noted in the quantitative responses, many preferred “complete control” (*“Flo,” 65*) over the decision-making process and “be[ing]in the driver’s seat” (*“Paul,” 81*) for as long as possible.

##### Family/Friend Involvement

Most respondents made at least one open-ended comment related to their desire for family/friend involvement (89%, 39/44). Commonly, respondents “want[ed] family members to definitely have a role” (*“Sue,” 70*), even if just to enact their pre-stated preferences. As one respondent stated, “my sons are in charge and they know all my rules and wishes that I won't deviate” (*“Tim,” 88*). In these instances, the role of family/friends was to advocate for a respondents’ pre-determined wishes. However, some respondents expressed comfort allocating decisional flexibility to their family/friends. For example, one participant stated they want their family/friends to “balance my wishes with their own” (*“Ellen,” 88*).

##### Treatment Care Preferences

Almost all respondents elaborated on their preferences for future care (93%, 41/44). These comments suggested that participants hoped to avoid treatment if it would result in pain, suffering, or becoming a burden. One stated, “I just don’t want to suffer” (*“Brock,” 79*), and a few hoped for compassionate EOL care from staff and “not just a worker following a health care book and doing what they choose” (*“Luke,” 88*). Open-ended comments on preferred death location added clarity to closed-ended responses. For example, of the 22 respondents who specified their preferred location of death in open-ended responses, 14 (64%) mentioned LTC, 2 (9%) mentioned a hospital, 3 (14%) suggested “it doesn’t matter” (*“Nora,” 88*), and 3 (14%) noted a preference for being at their or their relative’s home.

##### Post-Mortem Preparations

Although not specifically prompted in the workbook, most respondents referred to funeral or estate planning in their open comments (59%, 26/44). While many such comments broadly referred to “winding up financial affairs” (*“Max,” 89*) or “[finalizing] burial proceedings” (*“Jim,” 76*) some more specifically stated preferences for cremation, organ donation, or ensuring specific family members felt taken care of financially. In most cases, the framing of such preferences suggested that communication had already taken place (e.g., “my family already knows about my cremation plans”; *“Rob,” 89*).

##### Interpersonal Communication

While listing what matter most at EOL, respondents often expressed desire for interpersonal communications with family/friends (32%, 14/44). Desired communication ranged from resolving outstanding conflicts (e.g., “[to be] at peace with everybody”; *“Carly,” 68*) to expressing gratitude and saying goodbye (e.g., “I just want to thank [my loved ones] for doing their best to help me”; *“Martha,” 68*). In a few instances, respondents specified the context within which they wanted such communication to occur. For example, one wanted to “say goodbye to all family if possible—but not all at once” (*“Stuart,” 81*), suggesting the importance that this communication is done individually rather than collectively.

##### Spiritual Preferences

Several respondents expressed religious, cultural, or spiritual preferences in their open-ended responses (14%, 6/44). Many of these concerns were related to receiving a prayer, blessing, or EOL ceremony from a spiritual leader. For example, one respondent whose friend is an Indigenous medicine woman stated that she wanted “[her] friend to help [her] put on native clothing” as a part of a traditional EOL drumming ceremony (*“Jean,” 70*). Others referred to receiving last rites or a biblical prayer by their religious leader.

#### Advance Care Planning Reflection and Communication

An overview of the four process categories and their associated open-ended responses can be found in Table 7.

**Table 7:** Advance Care Planning Reflection and Communication: Open Ended Workbook Responses

Category	Participants	Excerpt Examples
Reflection	43	
* Vague*	31	“I would want to know if [a particular decision] was best for me; I trust certain people; you never know what is going to happen” “[at the end-of-life] happiness [matters most]” “I think my [loved ones] know [the role that I want them to have]”
* Specific*	41	“Being able to recognize my child, if in the hospital receiving excellent nursing care & being able to say good-bye to the ones I loved & being comfortable [is what matters most]” “I remember my husband’s EOL and I don’t want to be in that situation, in an out of the hospital three times and it was unpleasant” “If I am totally useless, I would need to be guided in my choices because even though I am cognisant, we don’t know what I'll be. If I couldn't make up my mind as to what I wanted, I'd really need help deciding and planning from my sister”
Preparing for Communication	20	“[I want] information on medically assisted dying” “[When I have conversations with loved ones, I want to] ask their advice to me, when they know me well. Tell them I love them.” “[I want to] incorporate my daughter-in-law in the whole process”
Reflecting on Communication	24	“[after the conversation I felt] good because everyone knows what they gotta do” “This conversation written here has been difficult. I would prefer not to dwell on my experiences and feelings that make me sad here” “I would just like to continue the conversation with my sister”

##### Reflection

Nearly every participant expressed reflections in their open-ended responses (95%, 42/44). While many respondents included reflections warranting further clarification (70%, 31/44), most documented at least one actionable reflection (93% 41/44). Examples of vague preferences included desires for “nursing care” (*“Mary,” 76*) or “peace” (*“Jack,” 77*), which did not specify the type of nursing care nor the conditions that would bring peace. Examples of specific contemplations included desires to die “with a full stomach and a glass of wine” (*“Alex,” 59*); or to receive “lots of pain pills to go painlessly” (*“Rob,” 89*).

##### Communication

While only 45% (20/44) of participants planned to discuss preferences with family/friends or staff, slightly more suggested communication had already occurred (55%, 24/44). Seemingly, some were referring to events prior to workbook engagement (e.g., “honestly, I have been through this 3 times. My [power of attorney] knows everything…. everything is arranged”; *“Tracy,” 94*). However, others referred to communication that seemed to occur as a consequence of workbook engagement (e.g., “I feel good that what I want in now known”; *“Tim,” 88*). Another participant reflected that the workbook inspired her to extend communications beyond post-mortem plans and to take the step to say, ‘I love you’ to her daughter (*“Sue,” 70*).

## Discussion

Our findings suggest that self-directed ACP workbooks can elicit LTC residents’ reflections on a range of care preferences important to EOL planning. This study’s workbook encouraged reflections on preferred decisional-roles, treatment directions, familial/friend involvement, post-mortem planning, interpersonal communication, and spiritual preferences. Our findings further suggest that if clarified and communicated, workbook reflections may inform the provision of EOL care that is aligned with residents’ values and preferences.

A key finding emanating from our study relates to residents’ desired family/friend involvement in care planning and decision-making. While most residents preferred to be fully informed of their conditions and prognoses, less than half expected family/friends to apply their care preferences without deviation. This suggests that ACP programs primarily focused on developing advance directives do not address the needs of LTC residents, many of whom consider decisional flexibility to be an important component of their relative’s role [6]. It also suggests that the decisional discretion afforded to family/friends could be experienced as burdensome if discussion of roles, expectations, and care preferences is not initiated in advance.

Notably, while participants expressed mixed feelings about family/friends implementing current care preferences in the event of diminished capacity, most wanted to be involved in decision-making for as long as possible. This points to the need for practices that support shared or assisted decision-making for persons with diminished capacity [34]. Unfortunately, current LTC practices more typically position family/friends as substitutes rather than partners in EOL care planning [30, 31].

Open-ended responses highlighted other components of future care of importance to residents in LTC: interpersonal communication (e.g., saying goodbye or resolving conflict), spirituality (e.g., EOL rituals); post-mortem planning (e.g., funeral); compassionate care and burden avoidance. This points to the need for ACP practices in LTC to directly solicit psychosocially oriented reflections alongside more medical reflections. To this end, we suggest that tools such as *Your Conversation Starter Kit* directly elicit such reflections, as not doing so potentially restricts reflection on issues and values of high significance to residents in LTC. It is noteworthy that since the time of writing, a revised version of the workbook has been produced which includes more psychosocially oriented open questions. Adding ranked questions related to spiritual care, interpersonal communication, post mortem planning, and independence may go a long way in eliciting care preferences of high importance to residents in LTC. 

Slightly more than half of our study participants appeared to value quality over quantity of life. This finding contradicts the literature, which suggests that almost all older persons living with frailty predominantly value quality over quantity [30]. It is noteworthy that a sizeable minority also expressed concerns that they may not receive enough care at EOL. Hence while it is certainly possible that invasive treatments near EOL may be preferred by some residents [35], concerns about LTC staff’s capacities to attend to and alleviate EOL suffering may underlie such preferences [36]. It is therefore important for staff to clarify these preferences in order to explore whether they are related to residents’ distrust or values [37].

Our combined open- and closed-ended responses on preferred death location proved difficult to interpret, suggesting that the workbook anchors were inconsistently interpreted. For example, respondents who explicitly named LTC as their preferred death location in open-ended responses were split between indicating openness to dying in a ‘health care facility’ and ‘at home’ in their closed-ended responses. Further, some respondents who selected dying ‘at home’ in their closed-ended responses clearly preferred dying in a family home, rather than LTC, in their open-ended comments. Death location preferences would be more accurately captured if the following potential choices were offered: LTC, family/friend home, hospital, or other (please specify). This is an important adaptation, given that preferred death location is a critical component of ACP [30–32].

In terms of ACP engagement, our findings suggest that self-directed ACP workbooks may improve ACP knowledge and elicit actionable reflections that can be communicated to family/friends and staff. Many participants expressed readiness for discussing these reflections with friends/family members, and workbook responses suggested some had already done so. However, some reflections required further clarification before they could be enacted, and more participants had contemplated than had communicated their thoughts, preferences, and values. Previous work on the distribution of paper-based materials has suggested that, despite interest in having conversations, moving from reflection to communication is complicated by fears of negative reactions [38]. Patients identify concerns around imposing family burden, poor relational dynamics, and a lack of family as barriers to activating conversations [16]. Our findings support the argument that self-directed workbooks may require staff support to encourage movement from contemplation to action by preparing residents and family/friends to manage the emotional reactions of the other [39]. Staff could be empowered to offer this support through training and organizational cultures that encourage staff to engage in follow up conversations with residents following workbook distribution.

Given the critical role many residents hoped family/friends would play in decision-making and care, including family/friends in discussions with staff from the time ACP is first introduced may support movement from ACP reflection to communication [40]. Encouraging such exchanges would clarify the role family/friends play at EOL. It would also arm staff and family/friends the knowledge to provide EOL care that aligns with residents’ values and preferences [16, 38].

### Study limitations

Our findings should be viewed in light of four important limitations. First, while our study aimed to provide an overview of care preferences important to LTC residents, the Likert-type questions may have limited the scope of reflection. Given the importance of psychosocial issues to LTC residents, more psychosocial prompts should be infused in the workbook. Our study suggests that family/friend communication, spiritual preferences at EOL, funeral planning and thoughts about being a burden and care/compassion may go a long way in eliciting reflections of concern to residents in LTC. Second, while our findings offer evidence that the workbook activated ACP engagement, we cannot know the extent to which these processes were caused by workbook use. Third, our small sample size and limited geographical range (urban localities in one Canadian province) precludes us from exploring how gender and ethnic diversity may have impacted our study. Future research should examine the impact of divergent identities on ACP engagement. Fourth, because we relied on LTC home staff for initial recruitment we are unsure how many eligible residents were informed of the study and invited to participate. As such the study has potential selection bias limiting the generalizability of our quantitative results.

## Conclusion

Our findings suggest that self-directed ACP workbooks may be useful for eliciting a range of LTC resident care preferences. Infusing psychosocial prompts and developing follow up protocols wherein residents are supported in communicating their reflections may be critical next steps to improving ACP engagement in LTC, and ultimately supporting EOL care that is aligned with the values and preferences of residents.

## Data Availability

The datasets used and/or analysed during the current study are available from the corresponding author on reasonable request.

## Abbreviations

ACP: Advance care planning
LTC: Long-term care
EOL: End-of-life
CHESS: Changes in Health and End-Stage disease and Symptoms and Signs
SDM: Substitute decision maker
